# Retraction: Surgical Management for Thoracic Spinal Tuberculosis Posterior Only versus Anterior Video-Assisted Thoracoscopic Surgery

**DOI:** 10.1371/journal.pone.0220752

**Published:** 2019-07-30

**Authors:** The PLOS ONE Editors

After publication of this article [1], the authors requested retraction due to concerns about the reliability of the results. They explained that they had re-reviewed the data for the study and found that some patients whose data were used had a diagnosis of spinal spondylitis rather than spinal tuberculosis. The authors did not respond to the journal’s follow-up questions to provide further clarification on the diagnosis and treatment of the participants and how the errors affected the analysis reported in the article. Without these further clarifications, it remains unclear whether the overall results and conclusions reported in the article are reliable.

In addition, the authors did not respond to the journal’s post-publication requests for ethics approval documentation or for information about the availability of the underlying data for the study. The Data Availability Statement for the article says, "Relevant data are within the paper," but the underlying data are not included in the article.

The *PLOS ONE* Editors retract this article as these unresolved issues have implications for the reliability of the results and adherence of the article to the journal’s ethics standards and Data Availability policy.

Fig 3 of the published article [1] was replaced at the time of retraction to address an error in the figure.

The authors did not respond to the retraction decision.
